# Robust parahydrogen-induced polarization at high concentrations

**DOI:** 10.1126/sciadv.ado0373

**Published:** 2024-07-24

**Authors:** Laurynas Dagys, Martin C. Korzeczek, Anna J. Parker, James Eills, John W. Blanchard, Christian Bengs, Malcolm H. Levitt, Stephan Knecht, Ilai Schwartz, Martin B. Plenio

**Affiliations:** ^1^ NVision Imaging Technologies GmbH, Wolfgang-Paul Straße 2, 89081 Ulm, Germany.; ^2^ Institute of Chemical Physics, Vilnius University, Saulėtekio av. 3, Vilnius LT10257, Lithuania.; ^3^ Institut für Theoretische Physik and IQST, Albert-Einstein Allee 11, Universität Ulm, 89081 Ulm, Germany.; ^4^ Institute of Bioengineering of Catalonia, 08028 Barcelona, Spain.; ^5^ Quantum Technology Center, University of Maryland, College Park, MD 20742, USA.; ^6^ School of Chemistry, University of Southampton, Southampton SO17 1BJ, UK.; ^7^ Department of Chemistry, University of California, Berkeley, CA 94720, USA.

## Abstract

Parahydrogen-induced polarization (PHIP) is a potent technique for generating target molecules with high nuclear spin polarization. The PHIP process involves a chemical reaction between parahydrogen and a target molecule, followed by the transformation of nuclear singlet spin order into magnetization of a designated target nucleus through magnetic field manipulations. Although the singlet-to-magnetization polarization transfer process works effectively at moderate concentrations, it is observed to become much less efficient at high molar polarization, defined as the product of polarization and concentration. This strong dependence on the molar polarization is attributed to interference due to the field produced by the sample magnetization during polarization transfer, which leads to complex dynamics and can severely affect the scalability of the technique. We address this challenge with a pulse sequence that suppresses the influence of the distant dipolar field, while simultaneously achieving singlet-to-magnetization polarization transfer to the desired target spins, free from restrictions on the molar polarization.

## Teaser

Dipolar field from the nuclear spins can disrupt the polarization transfer at high concentrations unless it is decoupled.

## INTRODUCTION

The nuclear magnetic resonance (NMR), one of the most widespread spectroscopic techniques with a broad range of applications, extending from chemical analysis and drug discovery to medical imaging, is intrinsically limited by its low sensitivity. This limitation is rooted in the weak nuclear spin polarization in thermal equilibrium, typically amounting to a few parts per million (ppm). Thermal equilibrium polarization and detection can be improved by increasing magnetic field strength, which may not be easily achievable. A promising alternative to address the sensitivity challenge involves hyperpolarization methods, which can enhance nuclear spin polarization by orders of magnitude compared to the level at thermal equilibrium (*1*–*18*).

Parahydrogen-induced polarization (PHIP) (*8*–*18*) is a hyperpolarization method that offers a high level of polarization and fast throughput of polarized samples. PHIP involves an irreversible hydrogenation reaction between a substrate and para-enriched hydrogen (parahydrogen) gas, which is used to embed the nuclear singlet order of parahydrogen in newly formed product molecules. Upon completion of the reaction, the singlet order is then transformed into observable magnetization on a target nucleus using a variety of methods, e.g., coherence transfer by NMR pulse sequences or adiabatic transfer schemes (*15*–*28*). As a result, PHIP can generate samples with molar polarization, defined as the product of the spin polarization and the concentration of target nuclei, reaching reported values of around 50 to 100 mM for ^13^C in fumarate (*13*).

The NMR signal is proportional to molar polarization, which is a better figure of merit than polarization alone for many applications such as metabolic imaging or fundamental physics experiments, for which high polarization alone is insufficient and high target concentrations are also desired (*13*, *14*, *29*). In addition, high molar polarization may unlock applications that inherently benefit from high sample magnetization, such as microscale NMR (*30*, *31*) or the nuclear Overhauser effect methods in liquid samples (*4*–*6*, *15*). This motivates our inquiry to what extent achievable molar polarization can be increased.

In this context, high molar polarization can introduce adverse effects. For example, a sample of ^1^H water only yields about 3 mM of ^1^H molar polarization at 9-T magnetic field and room temperature (111 M ^1^H concentration at 0.003% polarization), but this is sufficient intrinsic magnetization to act back on the sample itself. After radio frequency (rf) excitation, such magnetization in a tuned rf coil induces a current that generates an additional transverse field that rotates sample magnetization out of phase and causes radiation damping (*32*, *33*). This typically leads to line broadening, phase distortions, and other effects often associated with ^1^H- and ^19^F-rich samples.

A less pronounced phenomenon that does not require coupling to a tuned coil emerges from the (small) nuclear spin contribution to the magnetic flux density of the sample (*34*–*42*). A cylindrical 100 mM sample of ^1^H spins at 50% polarization (50 mM molar polarization) can generate a magnetic flux density of 180 nT corresponding to an 8-Hz resonance shift, while the previous example of water placed in a 9-T magnetic field would result in a 0.5-Hz shift (*38*–*40*). The backaction of these internal fields is known to induce chaotic dynamics even in highly symmetric, e.g., spherical, samples with uniform initial polarization distribution, as even minute inhomogeneities can be amplified rapidly (*7*, *39*, *40*, *43*, *44*).

**Fig. 1.**
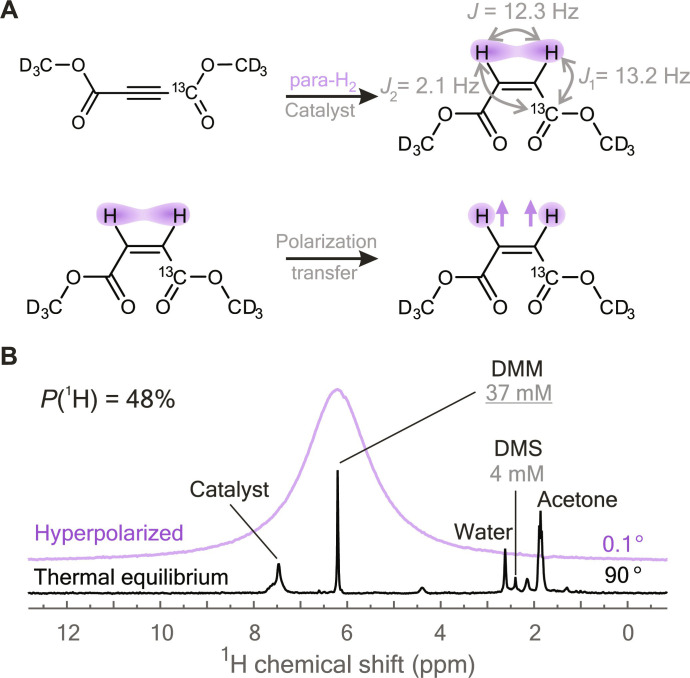
Hyperpolarization of [1-^13^C,d_6_]-dimethyl maleate (DMM) using PHIP. (**A**) Hydrogenation reaction of [1-^13^C,d_6_]-dimethyl acetylenedicarboxylate using parahydrogen yields DMM with two protons in a nuclear singlet state. *J*-couplings, taken from (*25*), are indicated. Deuterons and their couplings are ignored. The nuclear singlet state is transformed to magnetization of the protons using the magnetic inequivalence caused by nonsymmetric coupling to the ^13^C site. (**B**) The spectra of ^1^H hyperpolarized DMM (in purple) excited by a 0.1° rf pulse and of a thermally equilibrated sample (in black) excited by a 90° pulse. Spectral lines are assigned to DMM, catalyst, and other impurities; here, [1-^13^C,d_6_]-dimethyl succinate (DMS) is a secondary hydrogenation product. The polarization level of DMM is denoted by *P*(^1^H).

**Fig. 2.**
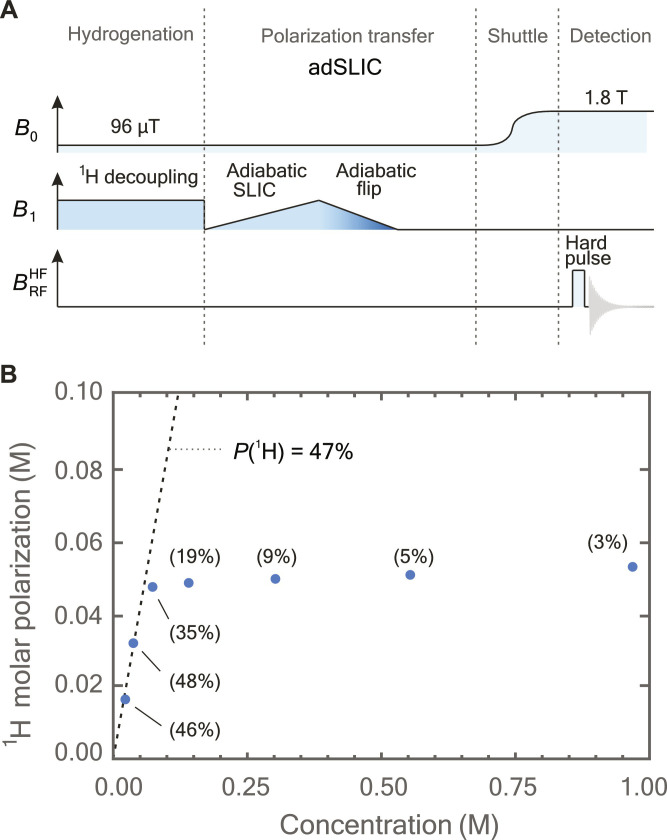
Hyperpolarization of DMM using the adSLIC protocol. (**A**) The magnetic field sequence used for the adSLIC polarization experiments. The procedure begins by hydrogenating a solution of [1-^13^C,d_6_]-dimethyl acetylenedicarboxylate at 96 μT and under continuous-wave irradiation at ^1^H Larmor frequency. Polarization transfer is performed by ramping up the amplitude of an on-resonant rf field (adiabatic SLIC). The magnetization is rotated to *B*_0_ by ramping down both the amplitude and frequency of the *B*_1_ field, with the frequency shift that is depicted as color shading. The sample is then transported to a benchtop NMR magnet [indicated as high field (HF)] where signal is acquired after a hard rf pulse. (**B**) ^1^H molar polarization of hyperpolarized target DMM as a function of concentration achieved by the adSLIC sequence. The amplitude sweep duration was set to 2 s. The dashed line represents a fixed polarization level of 47%, and polarization levels are shown in parentheses next to the data points.

Here, we show that this phenomenon, previously associated with the excitation of multiple echoes and experimental artifacts, can be sufficiently strong to interfere with polarization-transfer sequences in hyperpolarized samples. We obtained high ^1^H molar polarization using the hydrogenation reaction of [1-^13^C,d_6_]-dimethyl acetylenedicarboxylate with parahydrogen as shown in Fig. 1. This reaction produces [1-^13^C,d_6_]-dimethyl maleate (DMM) in which the two ^1^H spins from parahydrogen remain entangled in a nuclear singlet state but are no longer magnetically equivalent due to different *J*-couplings to the ^13^C site. The *J*-difference may be exploited by linearly ramping transverse magnetic field in resonance with ^1^H nuclei as shown in Fig. 2A. This method is known as adiabatic spin-lock induced crossing (adSLIC); it can induce complete conversion of singlet order to transverse ^1^H magnetization. In contrast to chemical shift–driven PHIP techniques such as ALTADENA or PASADENA, *J*-coupling–driven PHIP techniques may be operated at any bias field. This allows finding optimal conditions to avoid relaxation losses and thus offers practical advantages (*16*, *21*, *23*, *24*).

At low DMM concentrations (<100 mM), we consistently observe approximately 47% ^1^H polarization following the hyperpolarization process, a factor of ∼2 below the theoretical 100% limit, presumably because of the imperfect transfer from adSLIC, less than 100% para-enrichment, and losses due to spin relaxation. However, if the product concentration is increased beyond this value, the corresponding increase in molar polarization becomes highly nonlinear and reaches a limit at ∼60 mM of ^1^H molar polarization, as shown in Fig. 2B. Constant molar polarization independent of product concentration means that in this regime, the polarization is inversely proportional to the concentration of the polarized target. We hypothesize that this limit is due to a large dipolar field that emerges during the transformation of the singlet state into observable magnetization, which disrupts the adSLIC polarization transfer step. This is not a radiation damping effect, as the untuned and large excitation coil used for these low-field experiments couples too weakly to the nuclear spins to induce any appreciable radiation damping, and we have seen that a similar limit is encountered using magnetic field cycling, a simpler polarization transfer method not requiring a transverse (*B*_1_) field (*19*). Our observation presents a substantial obstacle for achieving high molar polarization and likely holds relevance for many other hyperpolarization techniques involving high sample concentrations or polarization, such as dynamic nuclear polarization or spin-exchange optical pumping (*1*).

Here, we propose a solution to overcome this challenge by implementing a Lee-Goldburg (LG) decoupling sequence that is commonly used in solid-state NMR to average out strong dipolar interactions (*45*, *46*). We explain how to combine this with suitable periodic modulation to re-establish a polarization transfer equivalent to adSLIC that we refer to as LG-adSLIC. In our experimental work, we verify the principle and demonstrate that the application of this pulse sequence leads to an order of magnitude improvement over the previous limit, yielding up to ∼450 mM ^1^H molar polarization. The achieved improvement is primarily limited by coil inhomogeneities in our device and can in principle be enhanced further. This should enable new PHIP applications involving high molar polarization such as microscale NMR (*30*, *31*) or the nuclear Overhauser effect methods in liquid samples (*4*–*6*, *15*) and may help to mitigate distant dipolar field effects in other areas of hyperpolarized NMR.

## RESULTS

We begin by considering the dipolar field generated in an ensemble of single spin-1/2 nuclei in the presence of off-resonant, LG decoupling (*45*, *46*). We may then easily extend our considerations to the case of a heteronuclear three-spin system incorporating polarization transfer during the said decoupling.

### Dipolar fields and LG decoupling

The Hamiltonian of an isolated single nuclear spin ensemble subject to external fields provided by magnetic coils and internal dipolar fields generated by the spin ensemble can be written as a sum of three terms

$$\begin{array}{cc} H_{I}\left(t\right) & =H_{0,I}+H_{\text{LG},I}\left(t\right)+H_{\text{DF},I}\\ & =-\gamma_{I}B_{0}I_{z}-\gamma_{I}B_{\text{LG}}\left(t\right)I_{x}+H_{\text{DF},I} \end{array}$$ (1)

where γ*_I_* is the nuclear gyromagnetic ratio, *B*_0_ is an external static magnetic bias field, *B*_LG_(*t*) is an externally applied transverse field oscillating at a frequency ω, and *H*_DF,*I*_ takes into account an internal magnetic field flux component due to all dipolar field contributions from distant nuclear spins.

Under most NMR conditions, the last term is negligible and can be ignored. At high concentrations or large polarization levels, however, dipolar fields can substantially affect the system dynamics. This interaction between each individual spin and the bulk of the sample is complex and may be described either microscopically, accounting for the dipolar interaction between all spins explicitly (*47*), or by adopting a mean-field description, which defines the dipolar field generated by a spatially homogeneous sample (*40*). For our purposes, these two descriptions yield equivalent results, and we use the mean field approach.

Expressing *H*_DF,*I*_ in the frame rotating at frequency ω of the continuous-wave transverse field *B*_LG_(*t*) and discarding rapidly oscillating terms give the state-dependent Hamiltonian [cf. equation 16 from (*48*)]

$$H_{\text{DF},I}^{\prime }=\Delta_{\text{DF}}\left[\langle \text{I}\rangle \cdot I-3\langle I_{z}\rangle I_{z}\right]$$ (2)

where $\langle I\rangle =\bar{\langle \psi \left(t\right)\left|I\right|\psi \left(t\right)\rangle }$ is the expectation value of the vector operator and the over-bar indicates an average over the spin ensemble. Assuming a spatially homogeneous sample

$$\Delta_{\text{DF}}=\Delta_{\text{DF}}\;\left(r_{l}\right)=\sum_{k\neq l}\frac{\mu_{0}\gamma_{I}^{2}}{4\pi }\frac{1-3{\left(e_{z}{\text{r}}_{\mathrm{kl}}\right)}^{2}/{|r_{\mathrm{kl}}|}^{2}}{{|r_{\mathrm{kl}}|}^{3}}$$ (3)

with ${\text{r}}_{\mathrm{kl}}={\text{r}}_{k}-{\text{r}}_{l}$ , where ${\text{r}}_{l}$ denotes the position of nucleus *l*. While in the general case $\Delta_{\text{DF}}\left({\text{r}}_{l}\right)$ depends on the position of spin *l* and of all the other molecules and their diffusive motion in the sample relative to spin *l*, the general structure of Eq. 2 remains independent of it. The contribution to $\Delta_{\text{DF}}\left({\text{r}}_{l}\right)$ from nearby spins is suppressed by molecular diffusion because Eq. 3 vanishes when ${\text{r}}_{\mathrm{kl}}$ is averaged over a spherically symmetric volume (*39*, *43*, *44*). Hence, only distant nuclei contribute to the dipolar field.

To minimize the influence of the dipolar field, we make the *B*_LG_ field off-resonant with respect to the Larmor frequency such that

$$\begin{array}{cc} -\gamma_{I}B_{\text{LG}}\left(t\right) & =2\omega_{\text{LG}}\text{sin}\theta \text{cos}\left(\omega t\right),\\ \omega & =\omega_{0,I}-\omega_{\text{LG}}\text{cos}\theta \end{array}$$ (4)

where ω_0,*I*_ is the Larmor frequency of spin *I* and the factor of 2 takes into account the average power of the linearly oscillating transverse field. The total Hamiltonian *H_I_* in the rotating frame then becomes

$$\begin{array}{cc} H_{\prime }^{I} & =H_{1,I}^{\prime }+H_{\text{DF},I}^{\prime }\\ & =\omega_{\text{LG}}\left(\text{cos}\theta I_{z}+\text{sin}\theta I_{x}\right)+H_{\text{DF},I}^{\prime } \end{array}$$ (5)

where ω_LG_ and θ define amplitude and orientation of a new effective field, respectively. The eigenbasis of $H_{1,I}^{\prime }$ leads to the tilted operators

$$\begin{array}{c} {\tilde{I}}_{x}=-\text{sin}\theta I_{z}+\text{cos}\theta I_{x},\\ {\tilde{I}}_{y}=I_{y},\\ {\tilde{I}}_{z}=\text{cos}\theta I_{z}+\text{sin}\theta I_{x} \end{array}$$ (6)

Rewriting the Hamiltonian in this basis and moving to a second interaction frame of *H*′_1,_*_I_* establishes what we henceforth refer to as the effective field frame. Neglecting rapidly oscillating terms, we find that the dipolar field Hamiltonian in this frame becomes

$$H_{\text{DF},I}^{\prime \prime }\left(\theta \right)=\Delta_{\text{DF}}\frac{\left(3{\text{cos}}^{2}\theta -1\right)}{2}\left[\langle \tilde{I}\rangle \tilde{I}-3\langle {\tilde{I}}_{z}\rangle {\tilde{I}}_{z}\right]$$ (7)

This vanishes at the magic angle $\theta_{M}=\text{arccos}\sqrt{1/3}$ . Note that the choice for θ_M_ coincides with the LG condition utilized in dipolar decoupling experiments in solids.

### Polarization transfer in the effective field frame

An extension of our off-resonant decoupling to singlet-to-magnetization transfer to achieve ^1^H magnetization in DMM (Fig. 1) may be given as follows. First, the total Hamiltonian of the coupled heteronuclear 3-spin system may be given by extending Eq. 1 to the modified form

$$H\left(t\right)=H_{\text{spin}}+H_{\text{rf}}\left(t\right)+H_{\text{DF}}$$ (8)

The dipolar field Hamiltonian *H*_DF_ inherits the same structure as *H*_DF,I_ by using the substitution $I_{i}\to I_{i}^{\Sigma }$ with $I_{i}^{\Sigma }≔I_{1,i}+I_{2,i}$ . Note that we do not consider corresponding terms from the *S* spins as these remain unpolarized throughout the experiment while merely experiencing a Zeeman shift from the *I*-induced dipolar field. This Zeeman shift does not contribute to the dynamics of the *I* spins. In the present case, *I* and *S* spins are ^1^H and ^13^C nuclei, respectively. Here, *H*_spin_ now includes Zeeman interaction for all spins *I* and *S* as well as *J*-couplings between them

$$\begin{array}{c} H_{\text{spin}}=H_{0}+H_{J}^{\mathrm{II}}+H_{J}^{\text{IS}},\\ H_{0}=-\gamma_{I}B_{0}I_{z}^{\Sigma }-\gamma_{S}B_{0}S_{z},\\ H_{J}^{\mathrm{II}}=2\pi JI_{1}\cdot I_{2},\\ H_{J}^{\text{IS}}=2\pi \;(J_{1}I_{1,z}+J_{2}I_{2,z})S_{z} \end{array}$$ (9)

The Hamiltonian *H*_rf_ describes the external transverse fields that are applied to *I* spins and is given by

$$H_{\text{rf}}\;(t)=-\gamma_{I}\left[B_{\text{LG}}\left(t\right)+B_{\text{mod}}\left(t\right)\right]\cdot I_{x}^{\Sigma }$$ (10)

The transverse field is now decomposed into two terms. The first term is the LG decoupling field *B*_LG_(*t*) as written in Eq. 4, which is used to mitigate the dipolar field by selecting the appropriate effective field angle. The singlet-to-magnetization transfer using adSLIC is performed during the said decoupling. Therefore, a second and lesser component *B*_mod_ is applied, which slightly modulates the decoupling field. The modulation field is given by

$$-\gamma_{I}B_{\text{mod}}\left(t\right)=-2\text{sin}\left(\omega t\right)\cdot 2\;\omega_{2}\left(t\right)\text{cos}\left(\omega_{\text{mod}}t+\phi \right)$$ (11)

where ω_mod_ is the modulation frequency and the time-dependent amplitude ω_2_(*t*) is needed for adiabatic polarization transfer. The second factor of 2 compensates for the linear polarization of the applied field.

Combining the terms and expressing the total Hamiltonian (Eq. 8) in the Zeeman interaction frame, we obtain

$$\begin{array}{cc} H\prime \left(t\right)= & H_{J}^{\mathrm{II}}+H_{J}^{\text{IS}}+H\prime_{\text{DF}}\\ & +\omega_{\text{LG}}\left(\text{cos}\theta I_{z}^{\Sigma }+\text{sin}\theta I_{x}^{\Sigma }\right)\\ & +2\omega_{2}\left(t\right)\text{cos}\left(\omega_{\text{mod}}t+\phi \right)I_{y}^{\Sigma } \end{array}$$ (12)

which simplifies with tilted operators in Eq. 6 to

$$\begin{array}{cc} H\prime \left(t\right)= & H_{J}^{\mathrm{II}}+H_{J}^{\text{IS}}+H\prime_{\text{DF}}\\ & +\omega_{\text{LG}}{\tilde{I}}_{z}^{\Sigma }\\ & +2\omega_{2}\left(t\right)\text{cos}\left(\omega_{\text{mod}}t+\phi \right){\tilde{I}}_{y}^{\Sigma } \end{array}$$ (13)

It is evident that the last two terms in this Hamiltonian describe the case of *I* spins being exposed to a static field of amplitude ω_LG_ and an oscillating transverse field with amplitude 2ω_2_. Therefore, if the modulating field is in resonance with the effective field such that ω_mod_ = ω_LG_, we can further simplify the Hamiltonian by expressing it in the doubly rotating frame and discarding rapidly oscillating terms. We find

$$\begin{array}{cc} H^{\prime \prime }\left(\theta ,t\right) & ={\tilde{H}}_{J}^{\mathrm{II}}+\text{cos}\theta {\tilde{H}}_{J}^{\text{IS}}+H_{\text{DF}}^{\prime \prime }\left(\theta \right)\\ & +\omega_{2}\left(t\right)\left(\text{cos}\phi {\tilde{I}}_{y}^{\Sigma }-\text{sin}\phi {\tilde{I}}_{x}^{\Sigma }\right) \end{array}$$ (14)

where the heteronuclear *J*-coupling Hamiltonian is scaled by the cosine of the effective angle. The tilde indicates the use of tilted operators retaining the structure of Eq. 9. For θ ≠ θ_M_, the dipolar coupling $H_{\text{DF}}^{\prime \prime }$ is partially suppressed compared to the original *H*_DF_ (cf. Eq. 2), whereas at the magic angle, we have $H_{\text{DF}}^{\prime \prime }=0$ and recover the dipolar field–free Hamiltonian. With the choice ϕ = 0, this leads to

$$H_{\theta_{M}}^{\prime \prime }\left(t\right)=\omega_{2}\left(t\right){\tilde{I}}_{y}^{\Sigma }+{\tilde{H}}_{J}^{\mathrm{II}}+\frac{1}{\sqrt{3}}{\tilde{H}}_{J}^{\text{IS}}$$ (15)

As a result of the LG decoupling, the adSLIC sequence achieving magnetization on *I* spins (^1^H in the present case) can be implemented in the effective field frame or exactly at LG frame via *B*_mod_(*t*) without obstruction by dipolar fields. As the derivation relies on the scale hierarchy ω_0_ ≫ ω_LG_ ≫ ∆_DF_, ω_2_, we use an adiabatic SLIC (*16*, *21*, *23*, *24*) to achieve robust transfer. It is important to stress that while the level anti-crossing condition for SLIC does not change (ω_2_ = 2π*J*), the transfer rate and, thus, adiabaticity are scaled by $1/\sqrt{3}$ as a consequence of the tilted effective field. This approach is also suited for implementing other homonuclear NMR sequences by selecting phase and time-dependent amplitude in Eq. 14.

### Experimental results

**Fig. 3.**
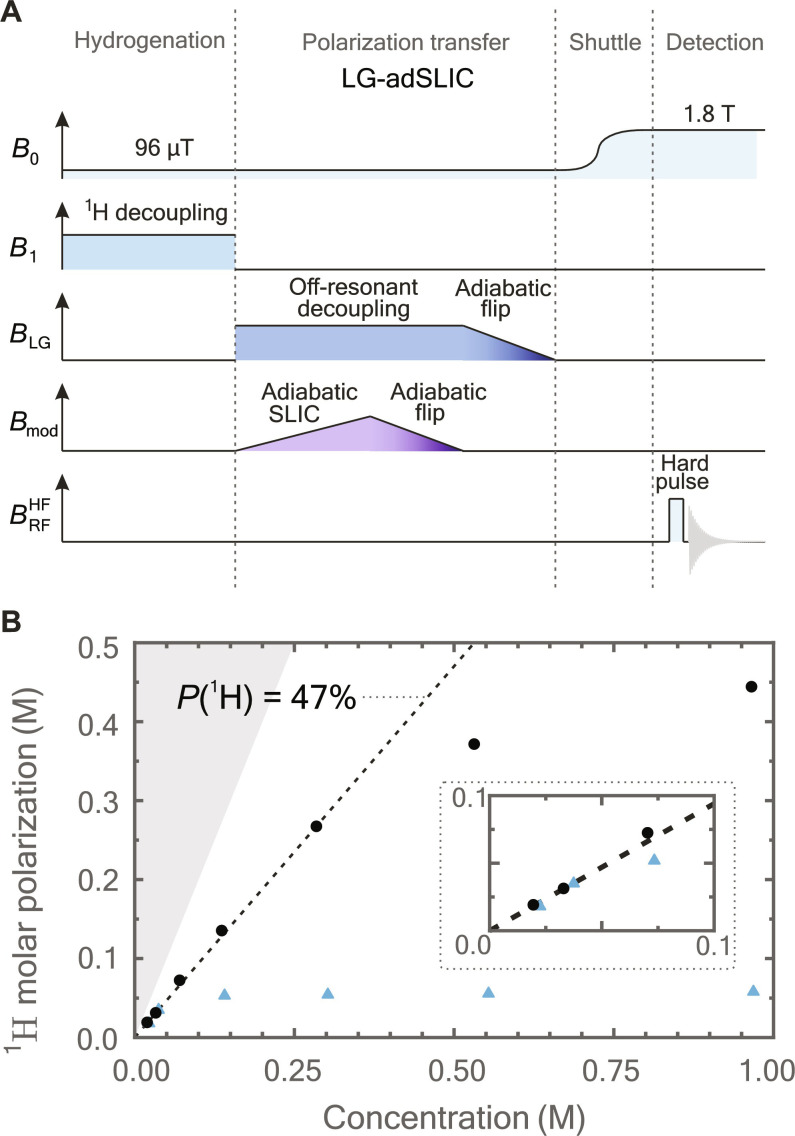
Hyperpolarization of DMM using the LGadSLIC protocol. (**A**) A modified magnetic field sequence LG-adSLIC that includes LG decoupling. Polarization transfer is performed with a modulation field (Eq. 11) mimicking the adSLIC transfer in Fig. 2A while under strong continuous irradiation with a resonance shift. To rotate the magnetization to align with *B*_0_, the amplitude and frequency of the *B*_mod_ field were first ramped down to rotate the magnetization along the effective field, and then the amplitude and frequency of the *B*_LG_ field were ramped down to rotate the magnetization along *B*_0_. (**B**) ^1^H molar polarization of hyperpolarized target DMM as a function of concentration achieved by the LG-adSLIC sequence (black dots) compared to the previous results when LG decoupling was omitted (blue triangles). The amplitude sweep duration was set to 2 s in both cases and the LG effective field amplitude was set to ω_LG_/(2π) = 600 Hz (more details in Materials and Methods). The shaded area indicates the nonphysical region in which ^1^H polarization exceeds 100%. A scaled inset plot is provided for clarity.

Introducing LG decoupling into the polarization process leads to a notable improvement in the achievable molar polarization at high sample concentrations. The experimental sequence and the results obtained with it are shown in Fig. 3. Operating under the same experimental conditions and contrasting the outcomes obtained by adSLIC and by LG-adSLIC, we find a strong indication that the limited molar polarization is not related to chemical impurities disrupting the polarization process. The linear scaling of molar polarization with product concentration (as indicated by the dashed line in Fig. 3) persists to a higher level of concentration when LG decoupling is used. There is still a decrease in sample polarization at molar polarizations above ∼300 mM, and we attribute this to insufficient LG decoupling at such high sample magnetization. In principle, this could be remedied by using a stronger LG decoupling field, but this requires higher *B*_1_ field homogeneity to ensure accurate matching of the LG resonance across the entire sample. This was impractical to implement on our equipment.

**Fig. 4.**
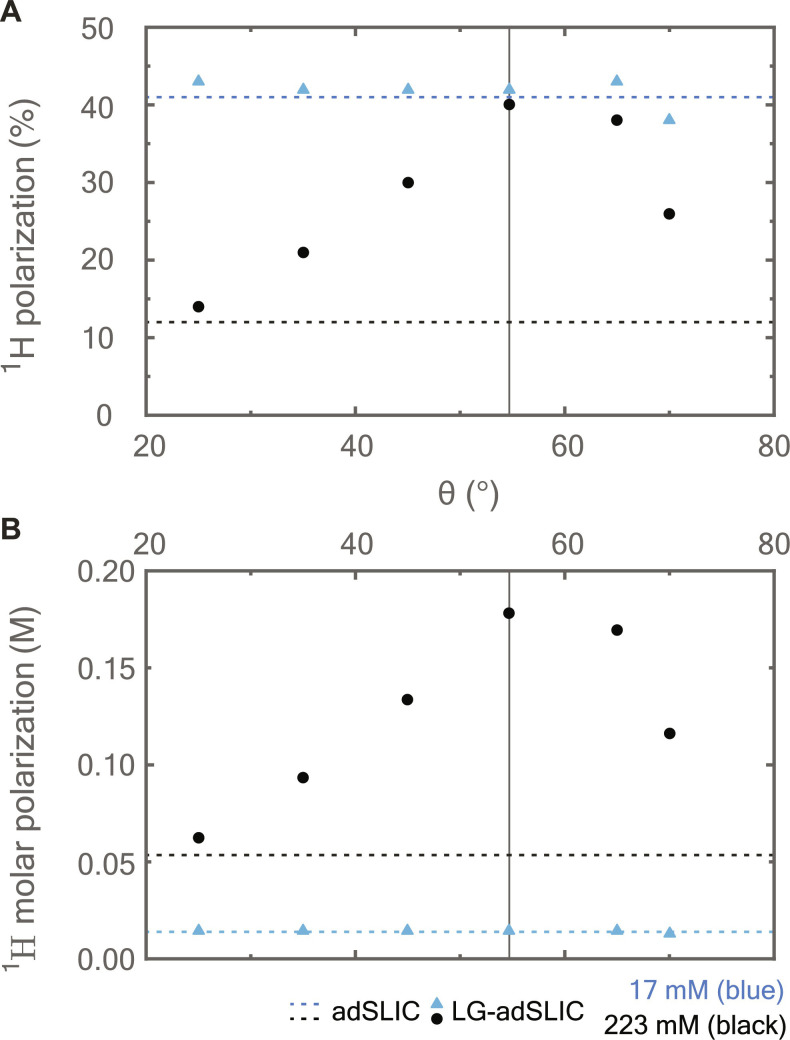
The geometrical aspect of the LG-adSLIC sequence. (**A**) ^1^H spin polarization and (**B**) ^1^H molar polarization of hyperpolarized DMM as a function of effective angle θ used in the LG-adSLIC sequence (Fig. 3A). Data points acquired at DMM concentrations of 17 and 223 mM are shown in blue and black, respectively. The amplitude sweep duration was set to 4 s and the effective field amplitude was set to ω_LG_/(2π) = 400 Hz (see Materials and Methods for more details). Dashed lines indicate the level of polarization acquired with the adSLIC sequence in Fig. 2A at high and low product concentrations. The magic angle value is shown as a vertical line.

The efficacy of LG decoupling on ^1^H polarization is investigated further by varying the effective field angle θ, and the results are shown in Fig. 4. At a low concentration of DMM (17 mM), no dependence on the angle θ was observed as the sample dipolar field is negligible and thus the LG decoupling does not affect the polarization. This was not the case at a higher concentration of DMM (223 mM) where LG decoupling is important for obtaining high polarization. The maximum polarization was achieved when setting the effective angle to the magic angle θ = θ_M_, which is consistent with prediction from Eq. 7. We reiterate that radiation damping is not expected to play a role in these experiments as the sample-coil coupling is negligible because low excitation frequencies were used and the large coil volumes result in a low filling factor.

## DISCUSSION

Here, we observe that the achievable molar polarization in PHIP-polarized samples is limited to approximately 60 mM, independent of the product concentration above a threshold of approximately 100 mM. This limit was observed in samples of DMM following the application of a low-field adSLIC sequence to induce ^1^H singlet-to-magnetization conversion. Our findings suggest that the limited molar polarization is due to a distant dipolar field originating from the polarized ^1^H spins as the sample becomes magnetized. The internal magnetic field along the cylinder axis in a sample of ^1^H spins at 60 mM molar polarization is approximately 214 nT, which would contribute 9 Hz to the Zeeman interaction. This value is comparable to the amplitude of the transverse field used and the spin-spin couplings in the molecule and thus disrupts the adSLIC pulse sequence. We have seen that a similar limit is encountered using simpler singlet-to-magnetization sequences such as adiabatic magnetic field cycling (MFC) as the bias field inducing the polarization transfer is in sub-microTesla regime as well.

To suppress this adverse effect, we implemented LG decoupling, leading to an improvement in the achievable molar polarization by an order of magnitude. Our work highlights those further improvements in hyperpolarization that can lead to circumstances where NMR pulse sequences can be disrupted by high internal sample magnetization and could complicate interpretation. Sequences that incorporate averaging of the dipolar interaction can help to reduce and diagnose this phenomenon. Such averaging properties are even more easily accessible in sequences designed to polarize ^13^C sites as the dipolar field scales quadratically with the gyromagnetic ratio of the polarized spins. This is crucial, as hyperpolarization methods that produce highly polarized solutions have become increasingly prevalent in recent years.

## MATERIALS AND METHODS

The precursor solution for DMM was prepared by dissolving 5 mM [Rh(dppb)(COD)]BF_4_ catalyst (CAS number: 79255-71-3) into acetone-d_6_. For the experiments with varied DMM concentrations, precursor concentrations were prepared in this order: 20, 40, 80, 160, 320, 640, and 1080 mM. Two precursor concentrations were used in Fig. 4, 20 and 300 mM for the blue and black points, respectively. Parahydrogen was produced by the Advanced Research Systems (ARS) parahydrogen generator packed with an iron monohydrate catalyst, running at 22 K temperature and producing gas with a para-enrichment level of ∼93%.

Figures 2 and 3 give an overview of the experimental protocol including the external magnetic fields experienced by the sample as a function of time. The experimental setup is described in (*16*) and comprises magnetic shield, solenoid, and an excitation coil. Because of a large coil volume and low Larmor frequencies, radiation damping is not expected to occur during the experiment. Each experiment starts by injecting 500 μl of solution into a tube and bubbling para-enriched hydrogen gas through the solution at 10 bar pressure at a bias field of 96 μT. This is followed by nitrogen bubbling at 10 bar to stop the reaction proceeding further. To avoid fast singlet order decay, resonant ^1^H decoupling is provided throughout the entire bubbling period, which, in all experiments, was fixed to 30 s (*16*, *17*).

Polarization transfer was performed following two different protocols as displayed in Figs. 2A and 3A. The first one consisted of a transverse field swept up from 0 to (2π) 25 Hz in amplitude (with respect to ^1^H), followed by an adiabatic flip pulse. The flip pulse was arranged by ramping the transverse field amplitude down in 1 s with a gradual carrier frequency shift (ω_0_ + ∆ω_0_) of ∆ω_0_/(2π) = −200 Hz. No decoupling was applied during the polarization transfer.

The second method included an off-resonant (LG) decoupling (cf. Eq. 4) during the polarization transfer to minimize the influence of the dipolar field. The effective field amplitude ω_LG_/(2π) was set to 600 and 400 Hz for experiments in Figs. 3 and 4, respectively. After the polarization transfer, a flip pulse was performed by ramping the transverse field amplitude down in 1 s with a gradual decoupling field frequency shift (ω + ∆ω) of ∆ω/(2π) = −200 Hz. Polarization transfer during LG decoupling was initiated by ramping modulation field (Eq. 11) amplitude from 0 to (2π)25 Hz (with respect to ^1^H). The modulation frequency was set to match the effective field amplitude (ω_mod_ = ω_LG_). To perform adiabatic pulse to flip magnetization along the effective field, the modulation amplitude was ramped down in 1 s with a gradual modulation frequency shift (ω_mod_ + ∆ω_mod_) of ∆ω_mod_/(2π) = −200 Hz. Two adiabatic flips are required to orient magnetization first along the *B*_LG_ and then along *B*_0_ fields before the sample is transported over to the NMR spectrometer.

The ^1^H free-induction decays were excited by a small flip angle pulse of (2π)20 kHz rf amplitude and recorded with 131,000 point density at a spectral width of 400 ppm. Additional ^1^H decoupling was used for all experiments. Thermal equilibrium ^1^H spectra were recorded at room temperature with a recycle delay of 90 s and with a 90° flip angle pulse. Polarization levels were calculated by comparing the ^1^H signals of hyperpolarized and thermally polarized samples. When estimating polarization level, the scaling factor of different excitation pulses was taken into account. The concentration of DMM was determined by comparing the thermal equilibrium signal to the signal of an external standard of known concentration measured under the same conditions. The molar polarization was calculated as the product of the concentration, the spin polarization, and the number of ^1^H sites in the molecule (two in the present case).
